# Comparative transcriptome analysis identified candidate genes involved in mycelium browning in *Lentinula edodes*

**DOI:** 10.1186/s12864-019-5509-4

**Published:** 2019-02-08

**Authors:** Seung-il Yoo, Hwa-Yong Lee, Kesavan Markkandan, Suyun Moon, Yong Ju Ahn, Sumin Ji, Junsu Ko, Seong-Jin Kim, Hojin Ryu, Chang Pyo Hong

**Affiliations:** 1 Theragen Etex Bio Institute, Suwon, 16229 Republic of Korea; 2 Department of Biology, Chungbuk National University, Cheongju, 28644 Republic of Korea; 3 Precision Medicine Research Center, Advanced Institutes of Convergence Technology, Suwon, Korea; 4 Department of Transdisciplinary Studies, Graduate School of Convergence Science and Technology, Seoul National University, Suwon, Korea

**Keywords:** Brown film, Cell wall degradation, Fruit body, *Lentinula edodes*, Light sensing, Mycelium, Transcriptome

## Abstract

**Background:**

*Lentinula edodes* is one of the most popular edible mushroom species in the world and contains useful medicinal components, such as lentinan. The light-induced formation of brown film on the vegetative mycelial tissues of *L. edodes* is an important process for ensuring the quantity and quality of this edible mushroom. To understand the molecular mechanisms underlying this critical developmental process in *L. edodes*, we characterized the morphological phenotypic changes in a strain, Chamaram, associated with abnormal brown film formation and compared its genome-wide transcriptional features.

**Results:**

In the present study, we performed genome-wide transcriptome analyses of different vegetative mycelium growth phenotypes, namely, early white, normal brown, and defective dark yellow partial brown films phenotypes which were exposed to different light conditions. The analysis revealed the identification of clusters of genes specific to the light-induced brown film phenotypes. These genes were significantly associated with light sensing via photoreceptors such as FMN- and FAD-bindings, signal transduction by kinases and GPCRs, melanogenesis via activation of tyrosinases, and cell wall degradation by glucanases, chitinases, and laccases, which suggests these processes are involved in the formation of mycelial browning in *L. edodes*. Interestingly, hydrophobin genes such as *SC1* and *SC3* exhibited divergent expression levels *in the normal and abnormal brown mycelial films, indicating the ability of these genes to act in fruiting body initiation and formation of dikaryotic mycelia. Furthermore, we identified the up-regulation of* glycoside hydrolase domain-containing genes *in the normal brown film but not in the abnormal film phenotype, suggesting that cell wall degradation in the normal brown film phenotype is crucial in the developmental processes related to the initiation and formation of fruiting bodies.*

**Conclusions:**

This study systematically analysed the expression patterns of light-induced browning-related genes in *L. edodes*. Our findings provide information for further investigations of browning formation mechanisms in *L. edodes* and a foundation for future *L. edodes* breeding.

**Electronic supplementary material:**

The online version of this article (10.1186/s12864-019-5509-4) contains supplementary material, which is available to authorized users.

## Background

*Lentinula edodes*, also known as the shiitake mushroom, is the third most commonly produced type of mushroom, following *Agaricus* and *Pleurotus*, and contributes almost 22% of the world mushroom supply [1]. The value of *L. edodes* in foods and medicines has recently been shown to derive from the physiological characteristics of this species, including anticancer, antiviral, antioxidant, antifungal, hypoglycemic and immunostimulating properties. With the potential as an effective bio-reagent for degrading lignocellulosic wastes, *L. edodes* also possesses biotechnological utility [2]. Due to these useful properties, the agricultural cultivation and biotechnological applications of this mushroom have greatly increased. The agricultural cultivation of *L. edodes* is normally achieved by two representative methods, namely, log-media-based and sawdust-media-based cultivation. The yield efficiency of the sawdust cultivation method is approximately 15% higher than that of the log cultivation method, and the sawdust cultivation period is short [3], thereby making it the preferred method for the cultivation of *L. edodes* worldwide.

Sawdust cultivation of *L. edodes* includes the vegetative mycelial growth process, the colonization of the growth substrate, brown film formation on the surface of the vegetative mycelial tissues, primordium initiation, and fruiting body development [4]. The formation of brown film on the mycelial tissue surface is a feature of sawdust cultivation for *L. edodes*, and this process is critical for maintaining water contents in the medium and ensuring a high quantity and quality of fruiting body formation. Additionally, the surface of the medium without brown film formation is easily occupied by pathogenic organisms, such as bacteria, green molds and fungi [3]. These results indicate that the presence of brown film on the sawdust medium surface provides a protective shield on the medium, similar to the bark of a tree [5]. Light signals have been well defined as essential factors for brown film formation [4, 6, 7]. The mechanism of light-induced brown film formation in *L. edodes* is linked to light perception, signal transduction pathways, and melanin pigment deposition [4]. Comparative transcriptomic and proteomic approaches to investigating the brown film of *L. edodes* mycelial tissues have revealed several important functional gene ontology (GO) classifications related to the formation of this film, including small molecule metabolic process, response to oxidative stress, and organic substance catabolic process classifications [4, 8]. However, comparative genetic molecular approaches for researching brown film formation with *L. edodes* mutants genetically defective in brown film formation have not yet been performed.

Edible basidiomycetous mushrooms have been bred with selection breeding, hybrid breeding by mating, biotechnological breeding using tissue culturing and protoplast fusion, and mutation breeding using radiation or chemical substances [9]. For the hybrid breeding methods, well-characterized genetic characteristics of parental strains, such as productivity, fruiting body traits, fruiting temperature, and developmental patterns, are mainly targeted [10]. Chamaram (strain # 07–84), a *L. edodes* strain, was developed as a suitable strain grown on sawdust media by the hybrid breeding method in South Korea. This strain displays a high productivity, with approximately 278.5 g of fruiting body per 1.3 kg in sawdust media and excellent fruiting body shapes [10]. With these attractive properties, the Chamaram strain is one of the most popular cultivated strains in south Korea. However, abnormal dark yellow partial brown film formation under light exposure during the early vegetative mycelial culture stage has been observed in this strain and has been reported as a major problem. The abnormal brown film formation increases susceptibility to pathogenic *Trichoderma* sp. and decreases the production of fruiting bodies, indicating that the Chamaram strain is an *L. edodes* mutant in mycelial brown film formation.

To obtain genomic resources for the improvement of genome-wide global genetic studies and useful agronomic traits, we recently completed the draft genome assembly of *L. edodes* [11]. The genome of *L. edodes* was estimated to be approximately 46 Mb in length, comprising 13,028 predicted gene models. Moreover, the application of PacBio long-read transcriptome data enhanced the accuracy of our gene set data with error correction and resulted in the final prediction of a total of 16,610 experimentally verified protein-coding genes [12]. The genomic resources obtained by our previous studies could be used for strengthening genome-wide analyses by contributing to the identification of targeted genes associated with a trait, transcriptome profiling, and comparative genomics. Such studies provide insight into unique features of the *L. edodes* genome. In the present study, we characterized the morphological phenotype changes of the Chamaram strain associated with abnormal brown film formation and compared the global gene expression patterns of brown film formation-related pathways.

## Results

### Light-induced phenotypic changes in *L. edodes*

**Fig. 1:**
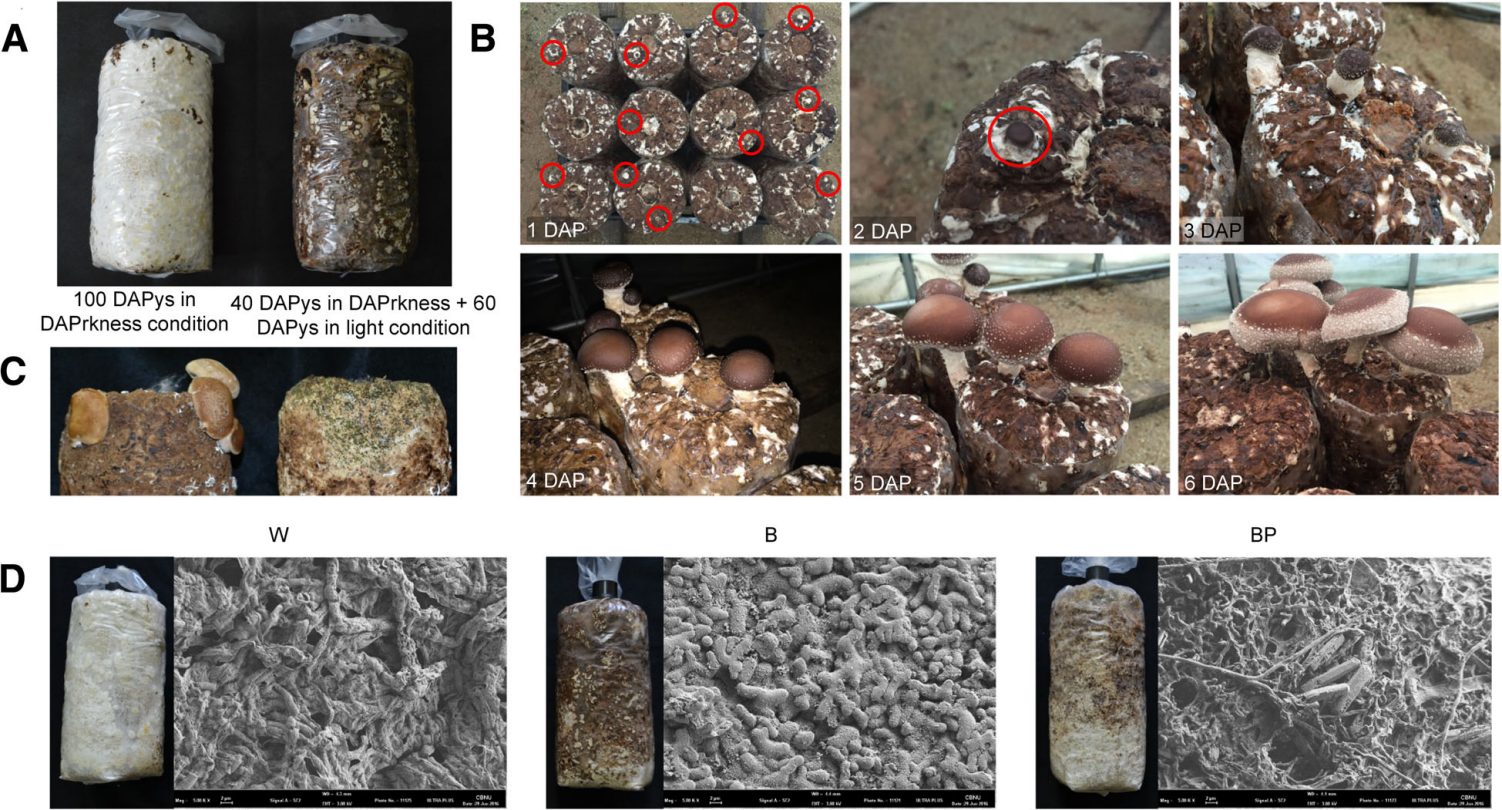
Brown film formation and fruit body development in the sawdust cultivation of the *Lentinula edodes* strain Chamaram. **a** Light-induced brown film formation on sawdust media. **b** Development of *L. edodes* fruiting bodies. **c** Fruiting body development and contamination by *Trichoderma* sp. on normal and dark yellow films formed by mycelial tissues. **d** Microstructures of white (W), normal brown (B), and partial brown (BP) film mycelium

The cultivation of *L. edodes* on sawdust media is divided into four phases: vegetative mycelial growth, light-induced brown film formation, primordium initiation, and fruiting body development. In general, the colonization of inoculated mycelium and the growth of vegetative mycelia on the sawdust media completed within 40 days in dark conditions; brown film formation was then followed by an additional 60 days of cultivation in the light (Fig. 1a). After mycelium browning on the sawdust media surface, the primordia were initiated within 5 to 6 days and then developed reproductive fruit bodies that could be harvested under suitable temperature and relative humidity conditions (Fig. 1b). We identified a Chamaram strain with an abnormal phenotype that generated dark yellow film on the sawdust surface due to early light exposure during the vegetative mycelial growth stage of *L. edodes*. The dark yellow film of this strain formed mycelial tissues that were defective in fruit body formation and were more susceptible than mycelia with normal brown film to contamination by other fugal pathogens, such as *Trichoderma* sp. (Fig. 1c). To investigate the more detailed molecular basis of brown film formation in response to light signals, we artificially induced three different types of mycelial tissue surface films, including vegetative white (W), normal brown film (B), and abnormal dark yellow partial brown film (BP), by a Chamaram strain by exposure to different light conditions, as described in the Methods section (Fig. 1d). Initially, the morphological hyphae of the W, B and BP mycelia were observed using a field emission scanning electron microscope. The browning surface on the sawdust media was densely arranged with elastic and plump hyphae. However, immature mycelial tissues consisted of slender hypha, and sawdust powder was also observed on the partially browning surface (Fig. 1d), suggesting that different growth and developmental processes were involved in the abnormal partial brown film formation of the mycelial tissues.

### Differential gene expression during mycelial browning

**Fig. 2:**
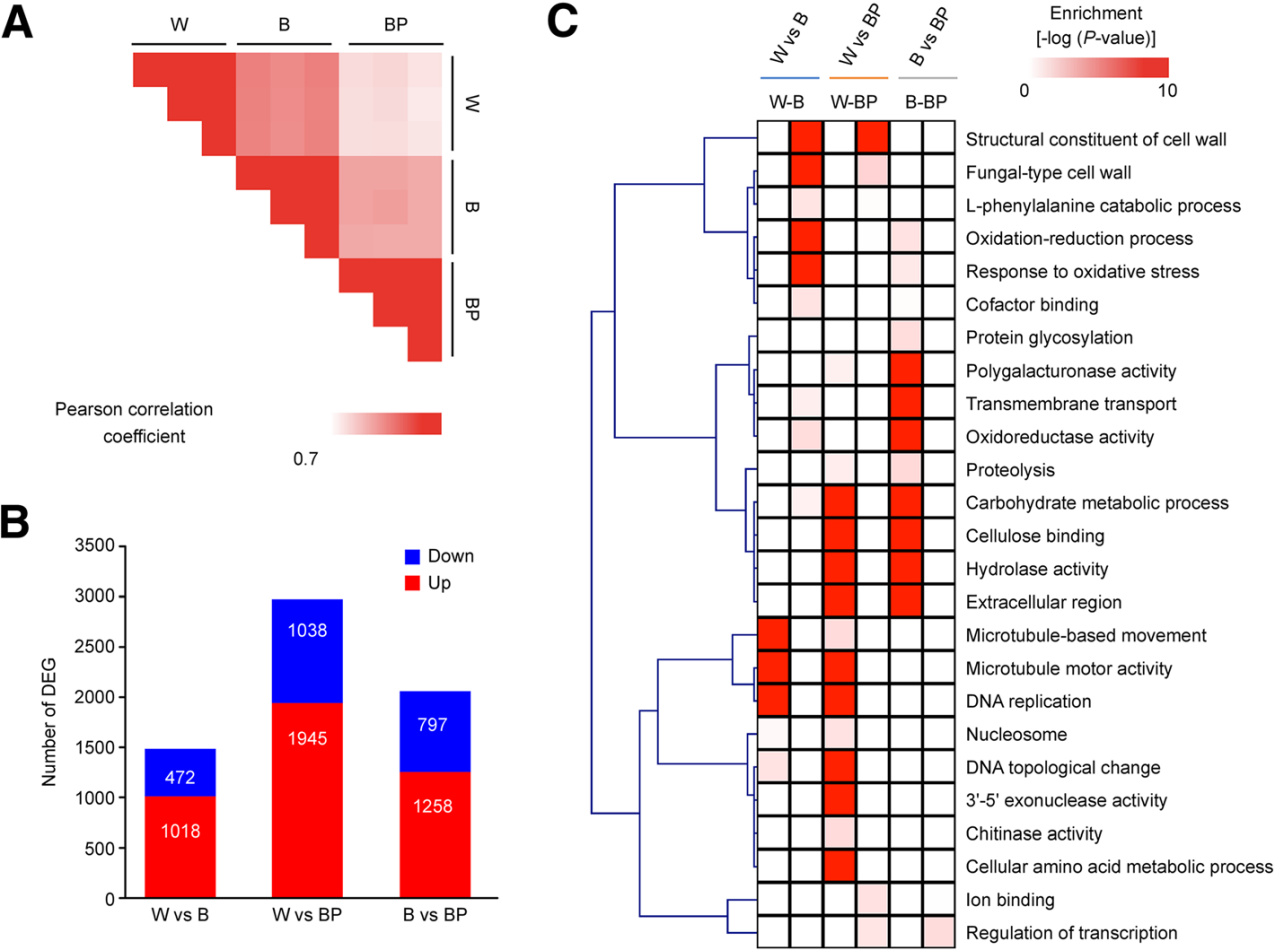
Overview of differential expression analysis for white (W), normal brown (B), and partial brown (BP) film mycelium. **a** Pearson correlation coefficients for pair-wise comparisons of the W, B, and BP mycelium transcriptome data. **b** Number of up- and down-regulated genes. **c** Gene set enrichment analysis (GSEA) of differentially expressed genes

To understand the genome-wide transcriptional changes underlying brown film formation-related developmental processes, we carried out RNA-seq analysis with the morphologically different W, B and BP mycelia. The average number of clean reads per sample was 20,797,944. After quality filtering, over 18.9 Gb of clean data was obtained from the nine libraries, and more than 99% of the high-quality clean reads were identified. Approximately 84% of the reads were mapped to the *L. edodes* draft genome sequence (Additional file 1: Table S1). Furthermore, the gene expression estimation was normalized with the value of fragments per kilobase of exon per million fragments mapped (FPKM), which revealed 16,581 known genes were expressed in all W, BP and B mycelium libraries (Additional file 2: Table S2). Consequently, significant differential expression was observed when the Pearson correlation coefficients between these phenotypes were calculated. Moreover, BP had more divergent expression levels than W and B (Fig. 2a). The above results reveal that our sample data were reasonable, with good correlations among biological replicates.

To identify the significantly differentially expressed genes (DEGs), the normalized read counts (FPKM values) of the W, BP and B mycelium phenotypes were statistically compared. Compared with W, 1490 DEGs were identified in B (1,018 up-regulated and 472 down-regulated genes), and 2983 DEGs were identified in BP (1945 up-regulated and 1038 down-regulated genes). Furthermore, when compared with B, 2055 DEGs were identified in BP (1258 up-regulated and 797 down-regulated) (Fig. 2b; Additional file 3: Table S3). The most DEGs were thus observed in BP, in which *L. edodes* showed abnormal dark yellow partial brown film (Fig. 1d), indicating that these DEGs may play key roles in *L. edodes* mycelial browning. For a better understanding of the DEGs involved in mycelial browning, the functional classes of DEGs were subjected to gene set enrichment analysis (GSEA). Our analysis revealed that the functions associated with cell wall (GO:0005618), oxidation-reduction process (GO:0055114), polygalacturonase activity (GO:0004650) and hydrolase activity (GO:0016787) terms were significantly enriched with down-regulated genes in the BP phenotype with light-induced mycelial browning. Remarkably, carbohydrate metabolic process (GO:0005975), DNA replication (GO:0006260) and cellulose binding (GO:0030248) GO terms were significantly enriched with up-regulated genes in both the W and B phenotypes (Fig. 2c; Additional file 4: Table S4). These results imply that light plays a significant role in the development and growth of *L. edodes*.

### Identification of light-induced phenotype-specific clusters

**Fig. 3:**
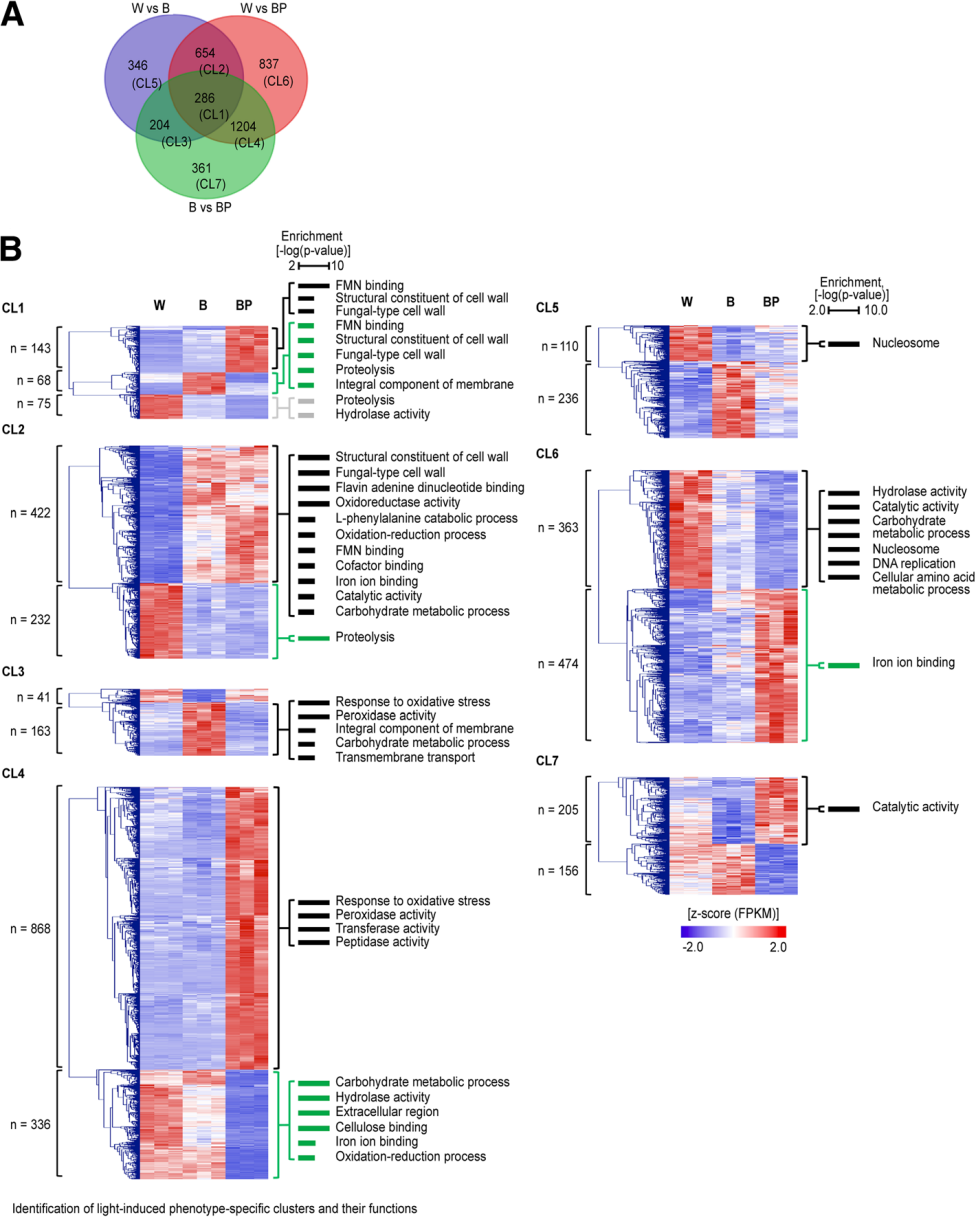
Light-induced phenotype-specific clusters and their functions. **a** Venn diagram presenting the overlap of differentially expressed genes among the three comparisons (W vs B, W vs BP, and B vs BP). **b** Hierarchical clustering heatmap of gene expression for each cluster and the representative gene ontology (GO) terms. The GO terms were analysed by GSEA (*P* < 0.01). Expression values (FPKMs) of genes were transformed to Z-score values.

To identify the phenotype-specific clusters, the DEG overlaps among the three comparisons were identified, and the functional categories of each cluster were analysed using GSEA (Fig. 3a, b; Additional file 5: Table S5). These results show the light-induced phenotype-specific clusters (CLs) and the associated functions of these CLs. CL1 shows distinct phenotype-specific expressions. In the B and BP groups, cell wall-related and light sensing-related genes were enriched. In the W group, genes associated with proteolysis and hydrolase activity were enriched (Fig. 3b). CL2 showed light-induced browning-specific gene expression. Genes associated with cell wall, light sensing, and oxido-reduction functions were up-regulated in B and BP; however, similar to CL1, proteolysis-related genes were down-regulated. CL3 and CL4 indicate the unique functions of normal and abnormal brown mycelium. Genes associated with responses to oxidative stress, peroxidase activity and transport exhibited phenotype-specific up-regulation. However, genes associated with carbohydrate metabolic processes were down-regulated in BP. These results indicate the functions of light-induced mycelial browning genes. Specifically, the formation of mycelial browning is involved in functions such as cell wall, light sensing, oxidoreduction, melanogenesis, and carbohydrate metabolic processes.

### Identification of genes related to mycelial browning in *L. edodes*

**Fig. 4:**
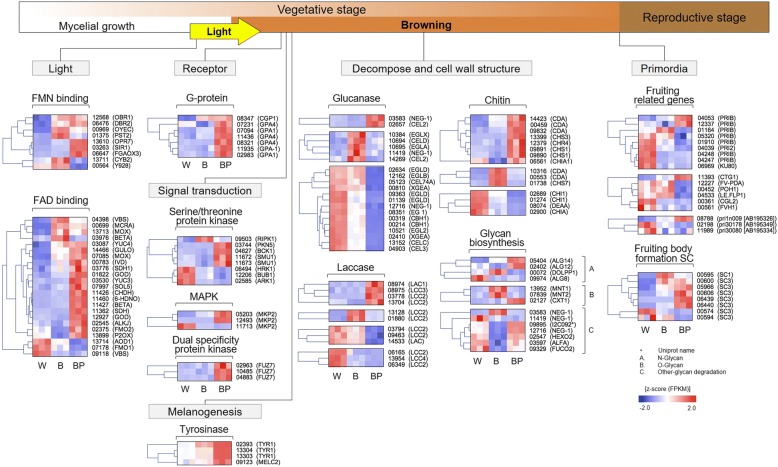
Genes involved in the regulation of mycelial browning in *Lentinula edodes*. The right side of the heatmap indicates the ID of the gene model of *L. edodes* and the homologous gene name. The gene expression values (FPKMs) were transformed to Z-score values

During the vegetative stage, light (blue light) induces mycelium browning. To identify genes specifically up-regulated during light-induced browning, we compared the W, B and BP phenotypes. Overall, we found that photoreceptors such as *FMN*-*binding* and *FAD-binding* genes and signal perception receptors such as G protein-coupled receptors (GPCRs) and their corresponding G proteins and kinases were significantly up-regulated in the BP phenotype and are therefore believed to be involved in the regulation of mycelial browning (Fig. 4; Additional file 6: Table S6). Interestingly, G proteins (*GPA1*; GENE07094 and *GPA4*; GENE11436) and kinase-encoding genes (*PKN5*; GENE03744*, BCK1*; GENE04627 and *SMU1*; GENE11672) were up-regulated, which indicates that these negative effectors might cause mycelial browning in the BP phenotype. The transcription factors involved in the MAP kinase signalling cascades *MKP2* (GENE11713) and *FUZ7* (GENE10485) were up-regulated. In addition, tyrosinase-encoding genes, *TYR1* (GENE02393, GENE 13304, and GENE 13303) and *MELC2* (GENE09123), that are known as oxidases, which are rate-limiting enzymes for the synthesis of melanin pigments, were also up-regulated in the B and BP phenotypes (Fig. 4; Additional file 6: Table S6). These results reveal that the aforementioned genes may induce pigmentation and sexual development during vegetative mycelium growth. Nevertheless, all tyrosinase-encoding genes were more highly enriched in the BP phenotype than in the B phenotype. This presumably indicates the existence of a negative feedback inhibition mechanism in light-induced melanin formation. This complex developmental programme requires more energy than simple vegetative growth, and vegetative mycelium seems to accumulate nutrients, which might later nurture developing fruit bodies during the reproductive stage (Fig. 4; Additional file 6: Table S6). Therefore, primary metabolism processes must adapt to use these sources. Hence, the complex light cycle may activate fungal cell wall glucanases, chitinases, and laccases encoding genes in B or BP. Our results show the phenotype-specific expression of these genes in the W, B and BP phenotypes. Remarkably, cell wall-degrading enzymes, such exoglucanases and endoglucanases (*NEG1*; GENE03583 and *CEL2*; GENE02657), were up-regulated in the B phenotype. Likewise, extracellular chitinolytic genes (*CDA*; GENE14423*, CHS3;* GENE13399*, CHR4*; GENE12379 and *CHS1*; GENE09891) were up-regulated in the B phenotype. These results indicate that glucanase and chitinase play a major role in *L. edodes* cell wall degradation during sexual reproduction (Figs. 1 and 4). Laccases, which are lignin-degrading enzymes produced by some fungi, catalyse the oxidation of phenolic substrates. We found the up-regulation of lignin degradation and detoxification genes (*LAC1*; GENE08974*, LCC2*; GENE06349 and *LCC3*; GENE08975) in both the B and BP phenotypes, which implies that laccases are important during mycelial browning. Finally, up-regulated fruiting body protein *PRIB* (GENE04248) and *SC3* (GENE00574) homologues were enriched in BP, probably indicating the initiation and formation of fruiting bodies. However, the *SC1* (GENE00595) homologue was up-regulated in W and B (Fig. 4; Additional file 6: Table S6).

### Distinct distribution of carbohydrate-active enzyme in light-induced mycelial browning

**Fig. 5:**
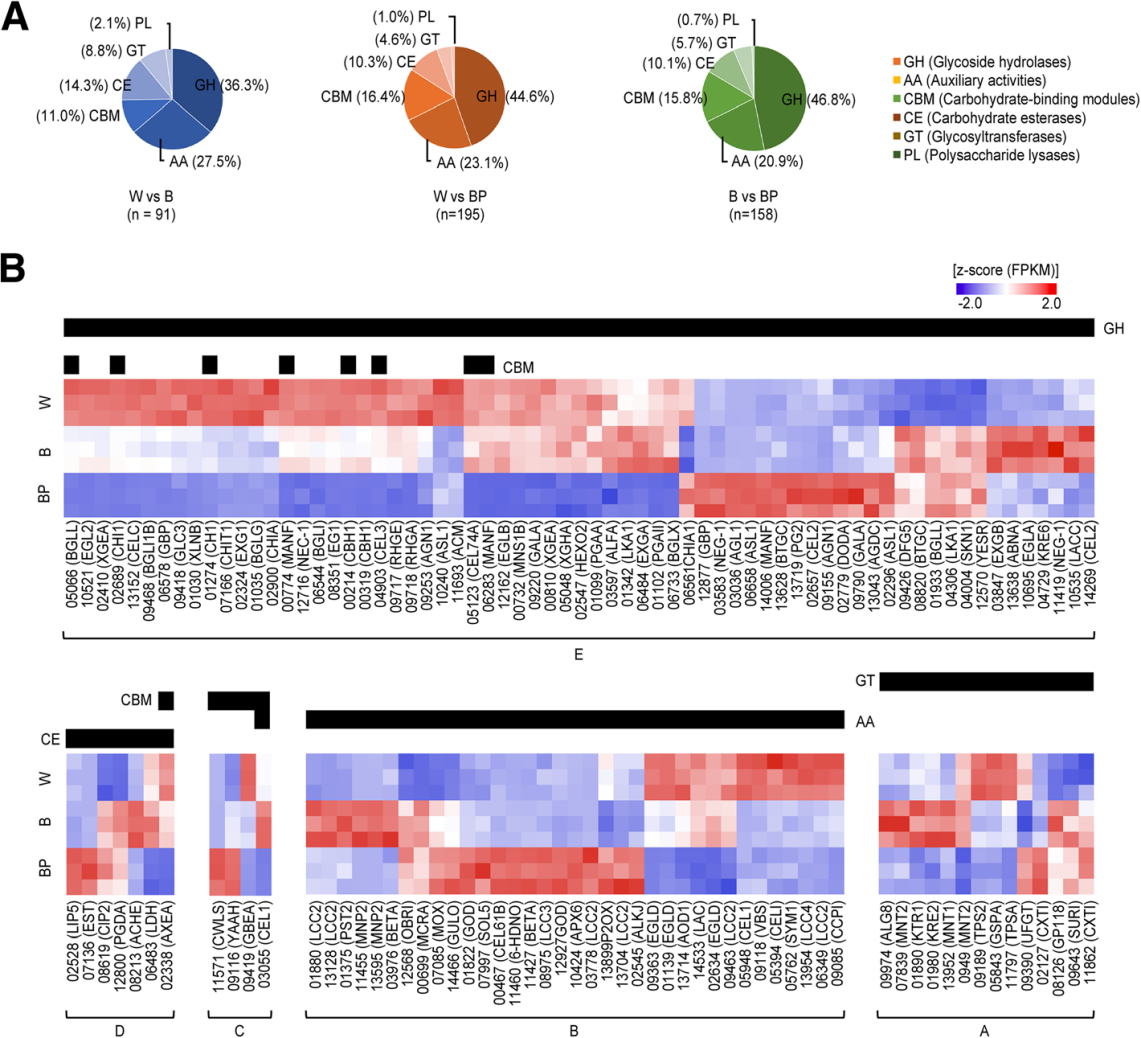
The distribution of carbohydrate-active enzyme-encoding genes identified from the differentially expressed gene sets (**a**) and the expression heatmap of these genes (**b**). The corresponding genes were searched using the carbohydrate-active enzyme database (CAZy) and were classified into primary domains such as glycoside hydrolase (GH), auxiliary activity (AA), carbohydrate-binding module (CBM), carbohydrate esterase (CE), glycosyl transferase (GT), and polysaccharide lyase (PL)

We also searched for families of structurally related catalytic and carbohydrate-binding modules of enzymes that degrade, modify, or create glycosidic bonds using CAZy [8]. Interestingly, when B and W were compared with BP, the highest distribution of DEGs were classified into superfamilies with glycoside hydrolase (GH), followed by auxiliary activity (AA), carbohydrate-binding module (CBM), carbohydrate esterase (CE), glycosyl transferase (GT) and polysaccharide lyase (PL) superfamilies, which appears to indicate the functional change associated with cell wall polysaccharide degradation (Fig. 5a). Our results show the divergent expression of genes associated with fungal cell wall decomposition (Fig. 5b; Additional file 7: Table S7). In particular, there were very few genes with similar expression patterns between B and BP. When mapping CAZyme-related DEGs to KEGG pathways, a high number of enzyme-encoding genes degrading cellulose and glucans were identified. The genes were up-regulated in B compared with W but were down-regulated in BP compared with W. In addition, genes associated with trehalose, maltose, and glucose synthesis were down-regulated in BP but were not differentially expressed in the B phenotype.

**Fig. 6:**
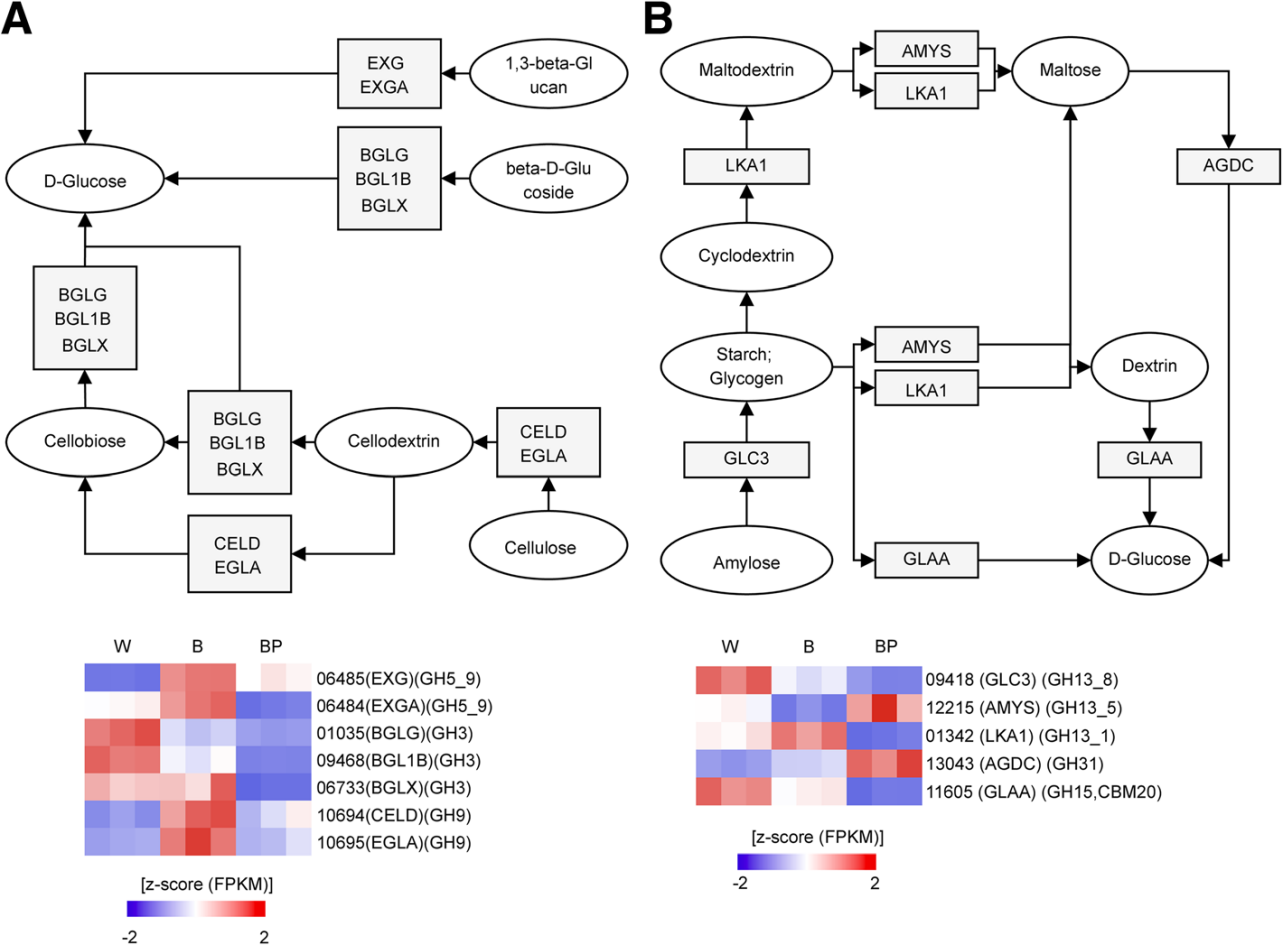
Gene expression changes in starch (**a**) and sucrose (**b**) metabolism

To verify the genes encoding enzymes involved in starch and sucrose degradation, we searched for the differential expression of genes involved in these metabolic processes. In the case of starch metabolism, four GH families containing potential β-glucosidases (*GH1 GH3, GH5* and *GH9*) were down-regulated in the BP phenotype. However, we could not find any α-glucosidases involved in starch metabolism (Fig. 6). In contrast, regarding sucrose metabolism the genes encoding α-glucosidase (*GH31*) and α-amilase (*GH13*) were up-regulated, whereas the α-glucoamylase (*GH15*) and N-terminal starch-binding modules (*CBM20*) were down-regulated in the BP phenotype. Furthermore, enzymes acting in the degradation of complex carbohydrates make the primary carbon sources for *L. edodes* growth and development unavailable.

### Validation of DEGs by qRT-PCR

To validate the RNA-seq data, the expression levels of selected DEGs, namely, *PST2*, *Sir1, LCC2A, MOX, Y928, LCC2B* and *priB2,* were quantified using qRT-PCR analysis (Additional file 8: Table S8). As presented in Additional file 9: Figure S1, the pRT-PCR data for these DEGs were basically consistent with the RNA-seq data. Linear regression analysis showed a positive correlation (*R*^2^ = 0.7365). In addition, the relative DEG expression levels were similar to those obtained from qRT-PCR.

## Discussion

### Formation of normal vegetative brown film of *L. edodes* in sawdust cultivation

The *Lentinula* mushroom genus is part of a family of white rot fungus that is naturally distributed in Asia, Australia, and the Americas [13]. *L*. *edodes* with edible mushrooms began to grow in China more than 800 years ago [14] and has excellent anticancer effects because it contains β-glucans such as lentinan [15]. Currently, this mushroom species is cultivated mainly in East Asian countries, such as Korea, China, Japan and Taiwan, and is an economically important edible mushroom that contributes approximately 22% of the global mushroom supply [1].

The cultivation of *L*. *edodes* mainly uses oak wood or sawdust. The log cultivation method requires a mycelial cultivation period of approximately 1 year. However, sawdust cultivation is favoured by farmers because it is possible to harvest fruiting bodies after approximately 100 to 120 days of mycelial cultivation. The sawdust cultivation method consists of 4 stages: mycelial colonization and growth in sawdust media, brown film formation in the light, primordium initiation, and fruiting body development [4]. These stages are divided into the vegetative stage until brown film formation and the reproductive stage after primordium initiation.

In this study, the *L. edodes* strain Chamaram formed dark yellow partial brown film (BP) under 100 days of light, vegetative white film (W) under 100 days of darkness, and normal brown (B) film under 40 days of darkness followed by 60 days of light. Therefore, we confirmed that early-stage incubation conditions are essential for normal browning formation during sawdust cultivation. Thus, darkness in the early stage of mycelial cultivation is necessary for normal brown film formation in *L*. *edodes* sawdust cultivation.

In sawdust cultivation, fruiting bodies generally occur after the formation of normal brown films on the surface of mature mycelia. In this study, the normal brown film (B) was more densely arranged with elastic and plump hyphae than the dark yellow partial brown film (BP) and the vegetative white film (W). The dark yellow partial brown film (BP) consisted of more immature mycelial tissues than the normal brown film (B) and the vegetative white film (W) observed on the sawdust powder. The dark yellow partial brown film (BP) was contaminated by *Trichoderma* sp. and produced fewer fruiting bodies than B. Therefore, brown film formation protected the sawdust media from other pathogens and was needed for primordia formation.

### Genes related to the light response in the vegetative stage

The brown film on the surface of the sawdust media in *L*. *edodes* sawdust cultivation was formed in response to light during the vegetative growth stage before primordia appeared. Brown film has been reported to be formed by the activities of light receptors, light signal transduction pathways, and pigment formation [4]. Light is a very important signal for every living cell [16]. In fungi, several developmental and physiological processes are regulated by light [17, 18]. Most fungi perceive blue light by using homologues of the white collar (*WC*) complex, a photoreceptor and transcription factor complex that was first found in *Neurospora crassa* [19]. The LOV-type PAS domain of *WC-1* is a photoreceptor that contains specific flavin binding motifs [20]. In this study, *FMN*- and *FAD*-binding genes and a flavin-type blue light photoreceptor gene [21] were expressed under three light conditions with the W, B and BP phenotypes. Therefore, the mycelium of *L*. *edodes* could have perceived blue light in any stage of growth.

After the introduction of blue light into the mycelium, the most important signal perception proteins are GPCRs and their corresponding G proteins. GPCRs are seven transmembrane pass proteins that bind stimuli in the extracellular space and transfer the information into the cell via conformational changes [22]. Therefore, GPCRs deliver signals from outside the cell into the cell. In this study, G protein genes were mainly up-regulated in BP, and *CGP1* was up-regulated in both B and BP. G protein genes were up-regulated only in BP and B that showed a phenotype change. The kinase genes involved in signal transduction were most up-regulated in BP, and the up-regulated kinase gene in B was *RIPK1*. Up-regulated *CGP1* and *RIPK1* genes that were not identified in BP, which showed an abnormal brown film phenotype, were identified in B. Therefore, the formation of normal brown film was assumed to involve *CGP1* and *RIPK1* after light perception.

In the light-induced phenotype-specific cluster analysis, genes related to cell wall, light sensing, and oxidoreduction functions were up-regulated in B and BP, but genes related to CL4 carbohydrate metabolic processes were down-regulated in BP but not in B. In fungi, light causes changes in carotenoid metabolism, polysaccharide and carbohydrate metabolism, fatty acid metabolism, nucleotide and nucleoside metabolism, and the regulation secondary metabolite production [16], and carbohydrates are mainly present as polysaccharides [23]. The transition from the vegetative stage in darkness to the reproductive stage in light may result in sudden metabolic changes [16], e.g., the polysaccharide content of the edible mushroom *Grifola frondosa* was higher in the primordia and fruiting body stage than in the mycelial stage [24]. Therefore, changes in carbohydrate metabolic processes in the *L*. *edodes* mycelium during the brown film formation period may affect fruit body development. In conclusion, the introduction of blue light results in browning on the mycelium surface and affects the development of fruiting bodies, which is related to carbohydrate metabolism.

Tyrosinase-encoding genes, such as *TYR1* and *MELC2*, in the brown film of *L. edodes* mycelium, were significantly up-regulated as compared with white film mycelium. This is consistent with the previous reports, which suggested that the activity of tyrosinase with light-enhanced expressions is strongly involved in the browning of mycelium and gill of fruiting body of *L. edodes* [25–29]. Interestingly, the two *TYR1* and *MELC2* homologs in the BP phenotype were significantly over-expressed compared to those of the normal B phenotype; 25.7-, 36.8-, 1.6-, and 2.3-fold changes in GENE13303 (*TYR1*), GENE13304 (*TYR1*), GENE02393 (*TYR1*), and GENE09123 (*MELC2*), respectively (Additional file 6: Table S6). This indicates that the extended exposure time to light, in particular blue-light wave length, may lead to such expression level and lead to abnormal, partial brown film formation of mycelium of *L. edodes*. This suggests the importance of appropriate exposure time of blue-light for a growth period in *L. edodes*. These speculations were further supported by observation of an unpredictable excess accumulation of light perception and cellular signalling-related components in BP (Fig. 4). In addition to phenolic compound for pigment formation in the gill of fruiting body of *L. edodes* [28, 30], phenylalanine ammonia-lyase (*PAL*) related genes, including GENE09131 and GENE09132, were also up-regulated (Additional file 2: Table S2), suggesting that *PAL* is co-ordinately involved in pigment formation with tyrosinase in mycelium browning. A blue-light photoreceptor *PHRA* (*WC-1* homolog; GENE06425) (Additional file 10: Figure S2), which is responsible for upstream functions of tyrosinase in the mycelium and fruiting body of *L. edodes* [26, 29, 31], presented different expression with that of tyrosinase (Additional file 2: Table S2; Additional file 11: Figure S3). In comparison with the expression level of *PHRA* in the W phenotype, the level in the B phenotype was slightly increased, and that in BP phenotype was decreased. This may suggest the unique function of blue-light photoreceptor to form normal brown film of mycelium. Our results indicate the key role of the melanogenesis process in the browning of *L. edodes* mycelium.

### Genes related to the light response in the reproductive stage

In fungi, light can regulate not only growth and pigment formation but also asexual and sexual reproduction [32]. In many conidial fungi, light pulses initiate conidation [16], and in some cultivated mushrooms, primordium formation requires light. In the case of *L*. *edodes*, exposure to continuous light by blue LEDs during the vegetative stage increases the productivity of the fruiting body [25]. In this study, hydrophobin genes such as *SC1* and *SC3* were expressed as genes related to fruit body development. Up-regulated *SC3* homologues were enriched in BP with no fruit bodies, and the *SC1* homologue was up-regulated in B, in which fruiting occurred. *SC1* and *SC3* are hydrophobin genes found in *Schizophyllum commune* [33]. *SC3* is expressed in both monokaryons and dikaryons, coats aerial hyphae with a hydrophobic layer, and mediates the adhesion of hyphae to hydrophobic surfaces [34]. *SC1* is activated only in the dikaryon and accumulates in the hyphal wall during fruiting body development [35]. Therefore, *SC1* related to fruit body development in the normal brown film was up-regulated under blue light.

Laccases are multi-copper-containing oxidases with diverse functions, including the polymerization/depolymerization of lignin, fungal pathogenesis, wound healing, sclerotization, morphogenesis, sporulation, pigmentation, fruiting body formation, melanin formation, and endospore coat protein synthesis [36]. A previous study showed that the laccase gene is involved in *Hypsizygus marmoreus* primordium initiation by increasing laccase activity and plays important roles in the differentiation of primordium into fruiting bodies [6]. In our study, the up-regulation of lignin-modifying genes (*LAC1*, *LCC2* and *LCC3*) showed clear evidence for lignin depolymerization and consequential cell wall decomposition in the BP phenotype. Likewise, various glycan-degrading proteins were up-regulated in the BP phenotype. Protein glycosylation is believed to play a critical role in various cell activities, such as the quality control of secretory proteins, cell wall integrity, environmental adaptation, antigenicity, and pathogenicity in some pathogenic fungi [37].

Lastly, we investigated the brown film formation process in *L*. *edodes* sawdust cultivation using a strain with abnormal brown film formation. The brown mycelial film on the sawdust media surface was formed by the introduction of blue light into mature mycelia cultured under darkness at the early stage of vegetative growth; therefore, G proteins and kinases were involved in this formation process. For the brown film that appeared on fruiting bodies, genes related to carbohydrate metabolic processes were involved in the development of the fruiting body, and *SC1*, which is related to fruiting body development, was up-regulated.

## Conclusions

Here, we identified candidate genes involved in light-induced mycelial browning in *L. edodes* by comparative transcriptome analysis of white, brown, and partially brown mycelium phenotypes. The formation of brown mycelium films is involved in functions related to, e.g., the cell wall, light sensing, oxidoreduction, and carbohydrate metabolic processes. This phenotype results from the formation of a normal fruit body with the up-regulation of *SC* homologues. CAZyme analysis showed a high distribution of DEGs with GH domains, which seemingly indicated a functional change associated with cell wall polysaccharide degradation. Interestingly, the expression levels of genes encoding glucans belonging to GHs, especially those in the exg family, were significantly changed, suggesting an involvement in lentinan biosynthesis. Our data enables the better understanding of browning formation in *L. edodes* and provides a foundation for future breeding.

## Methods

### Fungal materials

The *L. edodes* strain, Chamaram (strain # 07–84), was obtained from the Forest Mushroom Research Centre in Korea. To investigate the brown film formation characteristics in the mycelial tissue, the strain was cultured on oak sawdust medium (mixing oak sawdust and rice bran at a ratio of 4:1 (V:V)) to a water content of approximately 65% and a weight of 1.2 kg and was sterilized at 105 °C for 10 h. The sawdust media inoculated with Chamaram spawn were cultured under three conditions: (1) continuous darkness for 100 days to form the white film (W), (2) continuous darkness for 40 days and followed by under 16 h light/8 h dark cycle for 60 days to form the normal brown film (B), and (3) 16 h light/8 h dark cycle for 100 days to induce the partial brown film (BP) that was observed as a dark yellow film. After 100 days of culture, the strain on the surface of the sawdust media was sampled and observed for further analysis.

### Microstructure of browning surface

To observe the microstructure of the three different types of surface film on the sawdust medium, the mycelial samples of the surface were subjected to field emission electron microscopy. The samples were pre-fixed with 2.5% glutaraldehyde for 2 h and then washed with 0.1 M phosphate buffer two times for 10 min and post-fixed with 0.1% osmium tetraoxide for 1 h. After fixation, the samples were dehydrated with 15, 30, 50, 70, 80, 90, and 95% alcohol each for 15 min, and 100% alcohol with isoamylacetate for 10 min three times. The dehydrated samples were then coated with white gold for examination, and field emission scanning electron microscopy (LEO- 1530, Carl Zeiss, Oberkochen, Germany) was performed.

### RNA sequencing

For the RNA extraction, the strain on the surface of the culture medium (100 mg) was sampled, with 3 replications per condition. The samples were frozen in liquid nitrogen and ground into powder. RNA extraction was performed using an Easy-spin™ IIp Plant RNA Extraction Kit (iNtRON Biotechnology, Korea) following the manufacturer’s instructions. Approximately 35.7 μg (SD = ± 10.5) of total RNAs were extracted. The quality of the purified RNA was measured by an Agilent 2100 Bioanalyzer, following the manufacturer’s instructions (Agilent Technologies, CA); well-qualified RNA for next-generation sequencing (NGS) was defined as RNA with a RNA integrity number (RIN) of 7 and a 28/18 ratio of at least 1. All extracted samples revealed an RIN value of 9.1 ~ 9.6 and 28 s/18 s of 1.3 ~ 1.7.

Using 1 μg of the qualified RNA in each sample, poly(A) mRNA was enriched by magnetic beads with oligo (dT) and then sheared into short fragments. The cDNA was subjected to end-repair and poly(A) tailing and connected with sequencing adapters using a TruSeq Stranded mRNA Sample Prep Kit (Illumina, CA). The proper cDNA fragments, purified by a BluePippin instrument (Sage Science, MA) according to the manufacturer’s instructions, were selected for further PCR amplification. The final library sizes ranged between 350~450 bp. Subsequently, the libraries were subjected to paired-end sequencing with a 100 bp read length using an Illumina HiSeq 2500 platform, yielding an average of 21 million reads per library (Additional file 1: Table S1). The reads generated by the HiSeq2500 sequencer were then processed for quality with the following criteria: i) Discard the low-quality reads that contain more than 10% inaccurate bases (marked as ‘N’s), ii) Discard the reads with > 40% of bad quality bases (i.e., quality score < 20). The quality scores were analysed using the Fastqc tool (http://www.bioinformatics.babraham.ac.uk/projects/fastqc/) and the filtration process was performed using in-house scripts.

### Bioinformatics analysis

Clean reads were mapped into the draft genome of *L. edodes* [11] using Tophat [38]. The gene expression levels were then estimated using Cufflinks [39] based on the gene annotations. The option ‘-mask’ was applied to remove non-coding gene regions, and the options ‘-multi-read-correction’ and ‘fragbias-correct’ were applied to improve the estimation accuracy. All other options were set to the default values. DEGs between different developmental stages were analysed using Cuffdiff [39], with the gene information cutoff set at *p*-value < 0.01 and absolute fold change value set to ≥2. Hierarchical clustering for selected genes was conducted with MeV (http://mev.tm4.org) using the Euclidean distance and complete linkage method. All of the DEGs were searched against dbCAN (http://csbl.bmb.uga.edu/dbCAN/), which annotates the families of structurally related catalytic and CBMs (or functional domains) of enzymes that degrade, modify, or create glycosidic bonds [40]. GSEA was performed to examine the critical GO and KEGG pathways of the transcriptome. For this, the estimated expression levels of all genes were applied in GSEA and then the enrichment scores were calculated according to the ranked-ordered gene list. The KEGG pathways of the predefined gene sets were considered, and the pathways containing a minimum of 5 genes were evaluated. The significance scores were computed by 1000 nonparametric permutation tests [41].

### Quantitative qRT-PCR validation

Approximately 20 μl of 100 ng/μl total RNA from the mycelium was reverse-transcribed by TOPscript™ RT DryMIX (dT18 plus) (Enzynomics, Korea). The DEGs of interest related to blue light laccase and primordia formation were subjected to quantitative real-time PCR (qRT-PCR) analysis. The primers and gene information are listed in Supplementary Table S8. qRT-PCR was performed using Quant Studio 3 (Applied Biosystems, USA). The amplifications were performed using 1 μl (5 pM) of each specific primer, 10 μl of Power SYBR™ Green PCR Master Mix (Applied Biosystems, USA) and 1 μl cDNA in a final volume of 20 μl. The cycling parameters were 95 °C for 10 min followed by 40 cycles of 95 °C for 15 s, 58 °C for 25 s and 72 °C for 35 s. Each reaction was performed in biological triplicates using 18S as the internal control gene, and the relative gene expression was analysed using the 2-ΔΔct method. The analysed values were compared by the log2-fold change of the BP/W ratio.

## Additional files

Additional file 1: **Table S1.** Summary of the sequencing data utilized in this study. (XLSX 10 kb)

Additional file 2: **Table S2.** Summary of the transcriptome annotation and gene expression data. (XLSX 4025 kb)

Additional file 3: **Table S3.** List of the differentially expressed genes used for clustering analysis in each group. (XLSX 776 kb)

Additional file 4: **Table S4.** List of gene sets enriched in the differentially expressed genes. (XLSX 23 kb)

Additional file 5: **Table S5.** List of gene sets enriched in each cluste.r (XLSX 26 kb)

Additional file 6: **Table S6.** List of browning-related genes and their respective biological functions. (XLSX 42 kb)

Additional file 7: **Table S7.** List of differentially expressed carbohydrate-active enzymes (CAZyme) family genes. (XLSX 18 kb)

Additional file 8: **Table S8.** Primers and genes used for qRT-PCR. (XLSX 11 kb)

Additional file 9: **Figure S1.** Comparison of expression patterns by RNA-seq and qRT-PCR. The qRT-PCR log2 values (y-axis) were plotted as RNA-seq log2 values (x-axis). (PDF 7 kb)

Additional file 10: **Figure S2.** Multiple sequence alignment among *WC-1* (GenBank no. ESA41977), *PHRA* (GenBank no. BAF56991), and GENE06425. Multiple sequence alignment was performed by CLUSTALW. (PDF 94 kb)

Additional file 11: **Figure S3.** Expression of tryrosinases (A) and *PHRA* (B) in white (W), normal brown (B), and partial brown (BP) film mycelium. (PDF 10 kb)

## Abbreviations

CAZyme: Carbohydrate-active enzymes
DEG: Differentially expressed gene
GH: Glycoside hydrolase
GO: Gene ontology
GPCR: GPCRs G protein-coupled receptors
GSEA: Gene-set enrichment analysis
KEGG: Kyoto Encyclopedia of Genes and Genomes
qRT-PCR: Quantitative real-time polymerase chain reaction
