# Supplementary material for: Comparative transcriptome analysis identified candidate genes involved in mycelium browning in Lentinula edodes
Source: BMC Genomics. 2019 Feb 8;20:121. doi: 10.1186/s12864-019-5509-4 (PMC6368761; doi:10.1186/s12864-019-5509-4)
Supplement: Supplementary file 9 — Figure S1. Comparison of expression patterns by RNA-seq and qRT-PCR. The qRT-PCR log2 values (y-axis) were plotted as RNA-seq log2 values (x-axis). (PDF 7 kb) [file 12864_2019_5509_MOESM9_ESM.pdf]

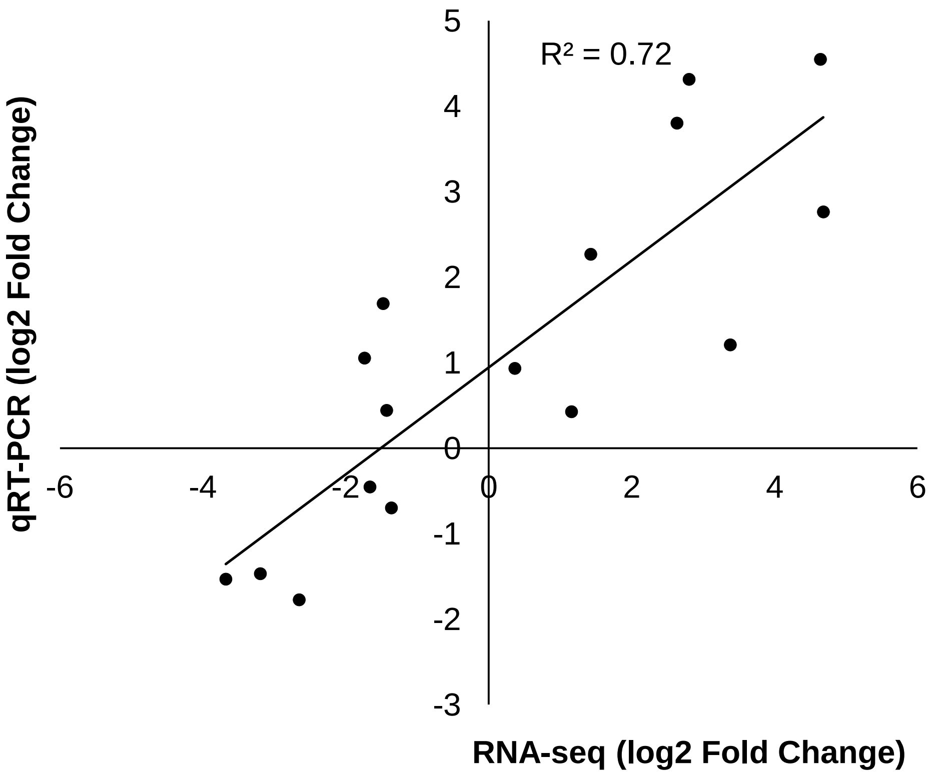

Supplementary Fig. S1. Comparison of expression patterns by RNA-seq and qRT-PCR. The qRT-PCR log2 values (y-axis) were plotted as RNA-seq log2 values (x-axis).
