# Supplementary material for: Comparative transcriptome analysis identified candidate genes involved in mycelium browning in Lentinula edodes
Source: BMC Genomics. 2019 Feb 8;20:121. doi: 10.1186/s12864-019-5509-4 (PMC6368761; doi:10.1186/s12864-019-5509-4)
Supplement: Supplementary file 10 — Figure S2. Multiple sequence alignment among WC-1 (GenBank no. ESA41977), PHRA (GenBank no. BAF56991), and GENE06425. Multiple sequence alignment was performed by CLUSTALW. (PDF 94 kb) [file 12864_2019_5509_MOESM10_ESM.pdf]

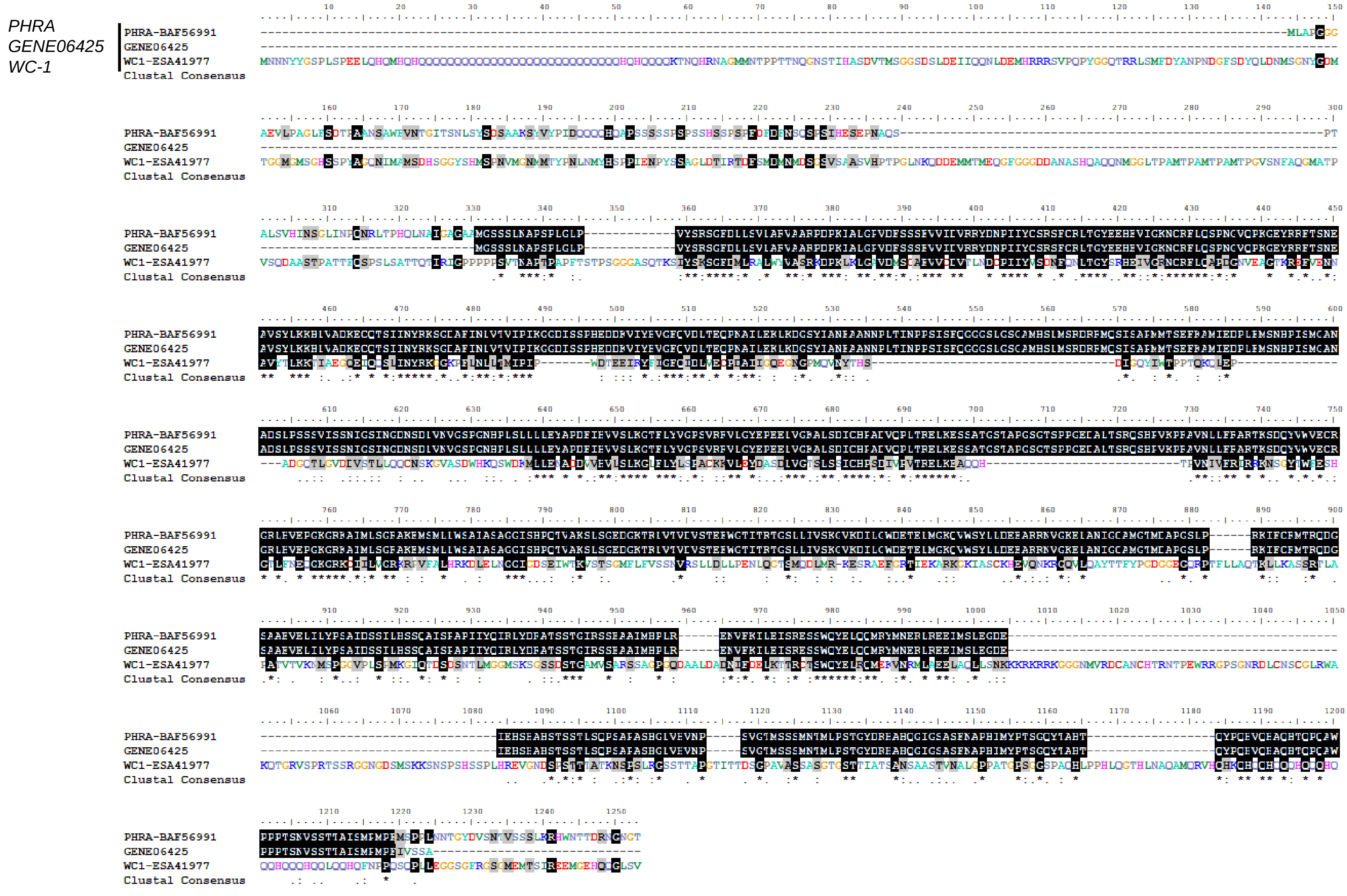

Supplementary Fig. S2. Multiple sequence alignment among *WC-1* (GenBank no. ESA41977), *PHRA* (GenBank no. BAF56991), and GENE06425. Multiple sequence alignment was performed by CLUSTALW.
