# Supplementary material for: Comparative transcriptome analysis identified candidate genes involved in mycelium browning in Lentinula edodes
Source: BMC Genomics. 2019 Feb 8;20:121. doi: 10.1186/s12864-019-5509-4 (PMC6368761; doi:10.1186/s12864-019-5509-4)
Supplement: Supplementary file 11 — Figure S3. Expression of tryrosinases (A) and PHRA (B) in white (W), normal brown (B), and partial brown (BP) film mycelium. (PDF 10 kb) [file 12864_2019_5509_MOESM11_ESM.pdf]

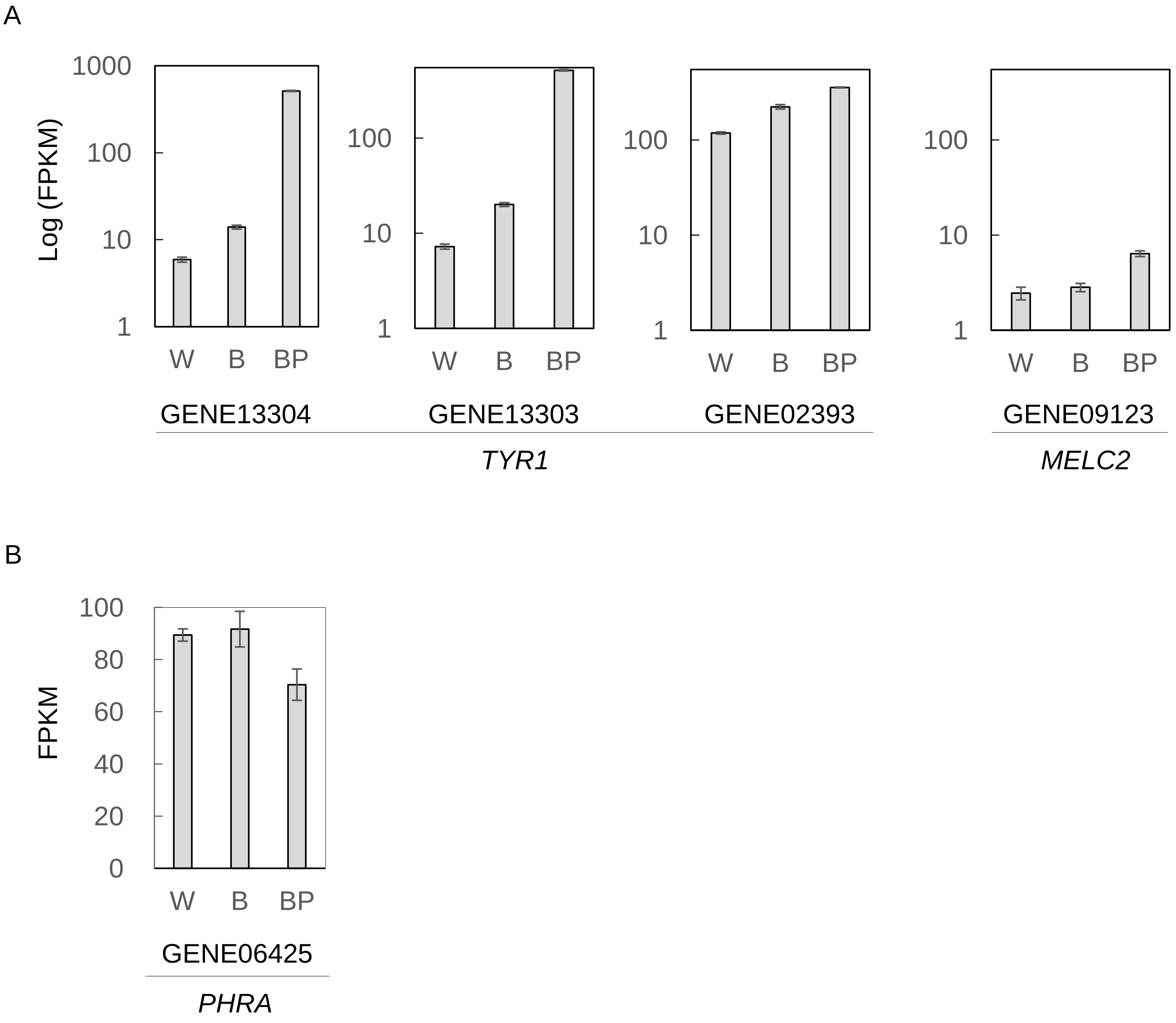


**Supplementary Fig. S3.** Expression of tyrosinases (A) and *PHRA* (B) in white (W), normal brown (B), and partial brown (BP) film mycelium.
